# ASSOCIATIONS BETWEEN LONELINESS AND COGNITIVE RESILIENCE TO NEUROPATHY IN OLDER ADULTS

**DOI:** 10.1093/geroni/igac059.1468

**Published:** 2022-12-20

**Authors:** Kathryn Jackson, Emily Wilroth, Jing Luo, Bryan James, Anthony Ong, David Bennett, Daniel Mroczek, Eileen Graham

**Affiliations:** Northwestern University, Chicago, Illinois, United States; Northwestern University, Chicago, Illinois, United States; Northwestern University, Chicago, Illinois, United States; Rush University, Chicago, Illinois, United States; Cornell University, Ithaca, New York, United States; Rush University, Chicago, Illinois, United States; Northwestern University, Chicago, Illinois, United States; Northwestern University, Chicago, Illinois, United States

## Abstract

Loneliness in the aging population is a concern, as increased loneliness is associated with decreased cognitive function and increased neuropathology. Less is understood about the relationship between loneliness and cognitive resilience. Cognitive resilience is defined as the discordance between a person’s actual and expected cognition given their neuropathology and can be estimated by extracting residuals from a model regressing cognition on neuropathology. Using data from two longitudinal aging cohorts (MAP/MARS), we estimated cognitive resilience proximate to death and cognitive resilience over time to use as the key outcomes. We then regressed these two cognitive resilience indicators onto loneliness level and slope. Higher baseline loneliness and increasing loneliness over time were both associated with lower cognitive resilience. Our results suggest that loneliness should be included into resilience-based prevention models, and interventions aimed at optimizing cognitive function across older adulthood should include loneliness reduction as a potential area of focus.

